# The feasibility and safety of simultaneous bilateral video-assisted thoracic surgery for the treatment of bilateral pulmonary lesions

**DOI:** 10.3389/fonc.2022.975259

**Published:** 2022-11-16

**Authors:** Xiandong Tao, Jiannan Zhao, Wei Wei, Zhengxiang Shan, Hongyang Zheng, Tiewen Pan

**Affiliations:** ^1^ Department of Thoracic Surgery, Third Affiliated Hospital, Navy Medical University (Second Military Medical University), Shanghai, China; ^2^ Department of Hepatobiliary Surgery, Third Affiliated Hospital, Navy Medical University (Second Military Medical University), Shanghai, China

**Keywords:** simultaneous bilateral surgery, video-associated thoracic surgery, bilateral pulmonary disease, feasibility and safety of surgery, thoracic surgery

## Abstract

**Background:**

The aim of this study was to evaluate the feasibility and safety of simultaneous bilateral video-assisted thoracic surgery (VATS) for the treatment of bilateral pulmonary lesions.

**Methods:**

The data of 11 patients who received simultaneous bilateral pulmonary surgery using VATS in the Department of Thoracic Surgery of The Third Affiliated Hospital of Naval Medical University between January 2016 and August 2021 were retrospectively analyzed.

**Results:**

The cases of four male and seven female patients, with a mean age of 57.54 ± 8.37 years (range, 44-67 years), were reviewed. Nonanatomic wedge resection, pulmonary segmentectomy or lobectomy *via* VATS were performed depending on each patient’s situation. Mean 1 second forced expiratory volume (FEV1) was 2.55 ± 0.66 L(range, 1.49-3.88 L), mean intraoperative bleeding volume was 91.81 ± 49.56 mL(range, 30-150 mL), mean operating time was 273.72 ± 68.98 min(range, 132-390 min), and mean drainage duration was 5.27 ± 3.60 days(range, 2-14 days), with a mean total drainage volume of 1,515.90 ± 772.75 mL(range, 530-3,225 mL). Only one postoperative complication (air leakage) occurred, with an overall complication rate of 9.09%. The mean postoperative hospital stay was 8.81 ± 3.60 days (range, 5-18 days), and the mean total cost of hospitalization was 67,054.53 ± 20,896.49 RMB (range, 47,578.45-123,530.8 RMB).

**Conclusions:**

Simultaneous bilateral pulmonary surgery using VATS for the treatment of bilateral pulmonary lesions is safe and feasible and can therefore be considered after strict preoperative evaluation of the patient.

## Introduction

It has been reported that lung cancer is the most commonly occurring type of cancer among new cases diagnosed and that it is the leading cause of cancer-related mortality in male patients (1). Fortunately, with the rapid development of imaging techniques as well as an increasing people’s health awareness, more and more pulmonary lesions are being detected in the early stage, especially in the case of carcinomas, some of which are bilateral.

Surgery is the mainstay of treatment for pulmonary carcinoma, being used both for the resection of tumors and to guide further therapy using pathology. Although staged surgery has been traditionally preferred for the treatment of bilateral pulmonary lesions, several institutions have reported the use of simultaneous bilateral pulmonary surgery; nevertheless, this surgical approach remains controversial due to its high level of invasiveness and risk of mortality (2, 3).

Currently, following the development of instruments and new surgical techniques, video-assisted thoracic surgery (VATS) is widely used in pulmonary surgery. It has several advantages, including shorter recovery time, decrease of postoperative complications and pain, and preservation of respiratory function (4, 5).The present study therefore investigated the feasibility and safety of simultaneous bilateral pulmonary surgery using VATS for the treatment of bilateral pulmonary lesions and summarized the experience of this treatment.

## Patients and methods

### Patients

The data of 11 patients who received simultaneous bilateral pulmonary surgery using VATS in the Department of Thoracic Surgery of The Third Affiliated Hospital of Naval Medical University (Eastern Hepatobiliary Surgery Hospital) between January 2016 and August 2021 were retrospectively analyzed. the included patients were consecutive. Demographic information, underlying diseases, surgical history, preoperative pulmonary function, surgical procedures, pathological status, pathological stage (8th edition of the TNM Classification of Malignant Tumors), intraoperative bleeding, operating time, drainage duration, total drainage volume, postoperative complications, postoperative hospital stay duration, and total cost of hospitalization were reviewed.

### Diagnosis

For all 11 patients enrolled in this study, chest computed tomography (CT) scans were performed to identify the size, location, number, shape, and likelihood of malignancy of the nodules or tumors. In order to exclude distant metastases, abdominal ultrasonographic examinations, bronchoscopy, brain magnetic resonance imaging, and positron emission tomography were performed in selected patients. Electrocardiograms and pulmonary function tests were routinely performed in all patients.

### Surgical procedures

Patients and their families were fully informed of the risks of simultaneous bilateral pulmonary surgery, and their consent was obtained. Endotracheal intubation was used for general anesthesia, and double-lumen endotracheal intubation was used to provide one-lung ventilation during the operation, so that the lung tissue on the operative side would collapse to provide a good view of the surgical field. The patient was placed in the lateral decubitus position with the surgical side facing up. After disinfection, the thoracoscopic single-port method/single-port method was used to perform the operation. A 3-cm incision was made in the fifth intercostal area of the midaxillary line as the observation and operation port. The single port method is generally used when the difficulty of surgery is expected to be high. A 1cm incision is made in the 8th intercostal area of the midaxillary line as the observation port, and a 3cm incision is made in the 4th or 5th intercostal area of the anterior axillary line as the operation port. After the operation, the drainage tube was placed, and the incision was closed. Then, the patient’s position was changed to remain in the lateral decubitus position, but with the unoperated side facing up. After sterilizing the towel, the thoracoscopic single-port surgery was performed. After the operation, the drainage tube was placed, and the incision was closed. We performed intraoperative frozen section examination for each specimen and adjusted the surgical method according to the intraoperative frozen section examination. The intraoperative frozen results were consistent with the final postoperative pathological results in all cases (6, 7). Surgical procedures were determined according to the size and location of the tumors as well as the pulmonary function of the patients. Nonanatomic wedge resection, pulmonary segmentectomy, or lobectomy, all using VATS, were performed depending on each patient’s situation. Additionally, if the diameter of the lesions, especially those with ground glass opacity, was less than 1cm, CT-guided puncture positioning was performed preoperatively in selected patients. Each tumor’s location was determined using intraoperative palpation so that, on the basis of negative margins, any normal pulmonary parenchyma could be preserved. In the case of segmentectomy, if intraoperative freezing indicated malignant lesions, the 12th lymph node group was dissected to determine the presence of lymphatic metastasis. In lobectomy cases, at least 3 sets of hilar and mediastinal lymph nodes were dissected if intraoperative freezing suggested malignant lesions. Sampling lymph node dissection was performed, and the most advanced TNM stage was considered the comprehensive stage. Regarding surgical resection priority, the side with the smaller lesion was usually resected first, in order to preserve pulmonary function for the next procedure. There were no adverse events related to the anesthesia (e.g., arrhythmias, hypotension requiring inotropes or vasoconstrictors, hypoxemia).

### Postoperative management

Postoperative management consisted of anti-inflammatory medications, expectoration, atomization, analgesia (morphine injection was used subcutaneously for pain relief within 3 days after operation, and non-steroidal analgesics were no longer used after 3 days), the promotion of sputum excretion, encouraging the patient to cough, and support for early ambulation. Bilateral single-cavity drainage tubes or a closed drainage tube and negative pressure suction were performed in selected patients based on their situations. At the side where lobectomy was performed, we indwelled the closed drainage tube to obtain full and guaranteed drainage. At the side where wedge, segment or lymph node resection was performed, we indwelled the single-cavity drainage tube with a smaller diameter because of less trauma and less blood and fluid leakage, in order to reduce the patient’s discomfort. We inserted drains in both hemithoraces, with the only difference being the size of the drain. These drains connected to underwater seal with adapters.

## Results

### General data

Ofthe11 patients enrolled in the study, four were male and seven were female, with a mean age of 57.54 ± 8.37 years (range, 44-67 years). Only two of the four male patients and none of the female patients had a history of smoking. Five patients had underlying diseases, including hypertension (three patients), diabetes(one patient), and chronic gastritis(one patient).

### Preoperative pulmonary function

Mean preoperative carbon monoxide diffusing capacity for single breath, DLCO SB was 7.92 ± 0.86 mmol/min/kPa(range,6.34-9.34 mmol/min/kPa), mean preoperative forced expiratory volume in one second (FEV1) was 2.58 ± 0.54L(range, 1.76-3.68 L), and mean preoperative FEV1% was 98.65 ± 14.91% (range, 78.00-130.10%), mean preoperative maximum ventilatory volume MVV was 94.26 ± 23L/min(rang, 61.22-137.47L/min). Data about the patients’ pulmonary function are given in **Table 1**.

**Table 1:** Detailed data of patients.

No.	Sex	Age	Smoking history	Underlying disease	DLCO SB (mmol/min/kPa)	FEV1 (L)	FEV1%	MVV (L/min)
1	F	53	0	N	7.39	2.6	98	98
2	F	45	0	N	8.21	2.72	89	104
3	F	54	0	N	7.34	2.45	87	97
4	F	44	0	Chronic gastritis	7.98	2.46	111.8	72.81
5	F	67	0	N	6.34	1.76	130.1	61.22
6	M	63	0	N	8.36	3.28	106	133
7	F	62	0	Diabetes	6.98	1.91	78	90
8	M	55	0	Hypertension	9.34	3.68	105.4	137.47
9	M	67	1	Hypertension	8.67	2.53	81.3	80.61
10	F	56	0	N	7.85	2.35	97.9	78.7
11	M	67	1	Hypertension	8.61	2.73	100.7	84.15
Total		57.54 ± 8.37			7.92 ± 0.86	2.58 ± 0.54	98.65 ± 14.91	94.26 ± 23.63

### Procedures and lesions

One patient underwent an upper lobectomy on the left side and lymph node biopsy on the right. Wedge resection was performed on at least one side in the other 10 patients, among which five underwent wedge resection on both sides. Right-side operations were performed first in five cases, with the principle of giving priority to the side with the smaller lesion. The surgeries in all cases were performed using VATS without conversion.

Pathologically, all malignant lesions were adenocarcinoma with or without invasion. Four cases had adenocarcinoma on both sides, one had benign lesions on both sides, and six had adenocarcinoma on one side and benign lesions on the other. Three patients were diagnosed with carcinoma *in situ* pathologically, six were staged as Ia, and only one was staged as IIIa. Data about the surgical procedures are given in **Table 2**.

**Table 2:** Detailed data about surgery.

No.	First side	Surgical procedure (R/L)	Pathology (R/L)	Stage	T	N	M
1	L	W/U S	Lymphoid tissue/Situ	Situ			
2	L	W/W	Situ Ad/AAH	Situ			
3	L	M Lob+Low W/U S	AAH/invasive Ad	Ia	1b	0	0
4	R	W/U S	Situ+infla/Infla	Situ			
5	L	W/W	Infla/MIA+Infla	Ia	1	0	0
6	L	W/W	Ad/Ad	Ia	1	0	0
7	L	U S+Lymph/W	MIA/MIA	Ia	1	0	0
8	R	U W+Low W/W	U Situ+Low situ/MIA	Ia	1	0	0
9	R	W/W	Infla/Infla	N	N	N	N
10	R	W/Low Lob+lymph	Invasive Ad/Invasive Ad	IIIa	1	2	0
11	R	Lymph/U Lob	Lymphoid tissue/Invasive Ad	Ia	1	0	0

N, None; L, left, R, right, U, upper lobe, M, middle lobe Low, lower lobe, W, wedge resection, S, segmentectomy, Lob, lobectomy, Lymph, lymph node dissection; Ad, adenocarcinoma; AAH, atypical adenomatous hyperplasia; Infla, Inflammatory nodule; MIA, microinvasive adenocarcinoma.

### Intraoperative and postoperative data

The mean intraoperative bleeding was 91.81 ± 49.56 mL (range, 30-150 mL), mean operating time was 273.72 ± 68.98 min (range, 132-390 min), and mean drainage duration was 5.27 ± 3.60 days (range, 2-14 days), with a mean total drainage volume of 1,515.90 ± 772.75 mL (range, 530-3225 mL). The mean postoperative hospital stay was 8.81 ± 3.60 days (range, 5-18 days), and the mean total cost of hospitalization was 67,054.53 ± 20,896.49 RMB (range, 47,578.45 to 123,530.8 RMB). Only one postoperative complication occurred, with an overall complication rate of 9.09%. This complication was air leakage on the left side, with the longest drainage duration of 14 days, the largest drainage volume of 3,225 mL, the longest postoperative hospital stays of 18 days, and the highest cost of hospitalization of 123,530.8 RMB. Intraoperative and postoperative data are given in **Table 3**.

**Table 3:** Detailed postoperative data.

No.	Operating time (min)	Intraoperative bleeding (ml)	Drainage duration (d)	Total drainage volume (ml)	Complication	Postoperative hospital stay (d)	Total cost of hospitalization (RMB)
1	311	50	2	610	N	8	64227.72
2	236	50	2	530	N	7	50464.54
3	300	150	6	1550	N	10	71690.69
4	390	100	4	935	N	7	47578.45
5	290	50	5	1750	N	7	52756.18
6	250	100	3	1425	N	6	52540.9
7	307	100	2	975	N	5	74279.79
8	210	80	6	1660	N	9	67372.27
9	132	30	9	2180	N	12	63030.53
10	249	200	5	1835	N	8	70128.04
11	336	100	14	3225	Air leakage	18	123530.8
Total	273.72 ± 68.98	91.81 ± 49.56	5.27 ± 3.60	1515.90 ± 772.75	1 (9.09%)	8.81 ± 3.60	67054.53 ± 20896.49

## Discussion

Bey Reuther first described multiple primary lung cancer in 1924 (8), and Martini and Melamed proposed its diagnostic criteria in 1975 (9). Surgical resection is still the most efficient treatment for multiple primary lung cancer, but this method remains controversial in the case of bilateral pulmonary tumors, with the indeterminacy of surgical procedures, extent of surgery, risks, and outcomes. The choice between simultaneous and staged pulmonary surgery has therefore become an important topic, and there has been no consensus regarding the treatment of bilateral pulmonary tumors until now.

Traditionally, a staged approach would be given priority in the treatment of bilateral pulmonary tumors, usually with an interval of between 15 days and 4 months (10, 11) between surgeries due to the decreasing risk of bleeding related to the extent of surgery and postoperative pulmonary complications, as well as the risk of anesthesia related to the duration of surgery (12). Some studies have reported cases of simultaneous bilateral pulmonary surgery, the potential advantages of which include lower overall risk and pain due to the need for only one instance of anesthesia and surgery, leading to faster postoperative recovery and earlier initiation of further treatment (such as adjuvant chemotherapy or targeted therapy) (10). In addition, a previous study found that simultaneous surgery meant that patients did not need to be hospitalized fora second surgery, thereby decreasing hospitalization costs (13), a finding that matches those of the present study.

Another study (14) compared the intraoperative changes between simultaneous and staged pulmonary surgery using VATS and found that unstable hemodynamics caused by longer operation durations and a change in position increased the use of vasoactive drugs and may also have increased blood loss. However, the study found no difference between the two types of surgery in terms of postoperative complications, thereby demonstrating the safety and feasibility of simultaneous bilateral pulmonary surgery with rigorous intraoperative management and suggesting that simultaneous surgery can be considered for patients with bilateral pulmonary lesions after strict preoperative evaluation.

Evidence for the feasibility and safety of simultaneous bilateral pulmonary surgery in the pediatric population was found in a Mayo Clinic (10) study in which 18 patients under the age of 18 underwent staged thoracotomy, nine patients under the age of age of 18 underwent simultaneous thoracotomy, and three patients under the age of 18 underwent both procedures. Compared with patients undergoing a staged thoracotomy, the mean hospital stay in the patients undergoing simultaneous thoracotomy was significantly lower(5.2 vs 10.6 days; P= 0.002), as was the duration of intensive care unit stay (1 vs 2 nights; P= 0.0001), days with tube thoracostomy (4 vs 8 days; P= 0.0005), and time to initiation of adjuvant chemotherapy (13 vs 30 days; P= 0.05). Furthermore, there were fewer postoperative complications in the group of patients undergoing simultaneous thoracotomy, although the difference was not significant (0 vs 3 events; P = 0.25).

Compared with a staged approach, simultaneous bilateral pulmonary surgery is accompanied by a greater risk of surgical injury, so greater attention should be given to its complications and mortality risk. The most common complications of pulmonary resection include pneumonia, air leakage, atrial fibrillation, and surgical site infection. In the present study, only one of the 11 patients experienced postoperative complications, and this complication (air leakage) occurred without pneumonia, atrial fibrillation, and surgical site infection, resulting in an overall complication rate of 9.09%, which was lower than the complication rate of 18.8% found in a similar study (13). Moreover, no postoperative death occurred in the present study, with a mortality rate of 0% in 90-day. However, changes in pulmonary function after the resection of pulmonary lesions should be a focus of postoperative monitoring due to the high incidence of cardiopulmonary complications, such as arrhythmia, anoxia, and respiratory failure, which can be caused by a significant loss of pulmonary function. It is therefore important to preoperatively evaluate pulmonary function and determine the extent of surgery required according to each patient’s situation.

It is well known that a decrease in volume of the pulmonary vascular bed and lung capacity may cause respiratory failure, thereby influencing postoperative morbidity and mortality. It has also been reported that poor pulmonary function is a predictor of morbidity and mortality after pulmonary resection (15). Therefore, evaluating pulmonary function inpatients who will undergo simultaneous bilateral pulmonary surgery is crucial. Previous research has demonstrated that pneumonectomy is safe if FEV1 is greater than 2L and that lobectomy is safe if it is greater than 1.5 L or if its predicted value is more than 80% (16, 17).For simultaneous bilateral pulmonary surgery, anFEV1/forced vital capacity of more than 75% is recommended (18).It is interesting to note that female patient shave been found to have better outcomes than male patients after pulmonary surgery, which may be caused by their smaller vital capacity (19).

The extent of surgery is also a key factor that can influence postoperative pulmonary function and thereby the occurrence of complications. Therefore, in order to ensure the safety of patients in the present study, wedge resection was performed in most cases (10/11, 90.90%) of simultaneous bilateral surgery to ensure enough residual pulmonary function to satisfy ventilation during surgery on the opposite side. Zeiher (20) found that the number of pulmonary segments removed can predict postoperative pulmonary function, and the relationship between the number of pulmonary segments resected and postoperative pulmonary function is of great significance, so segmentectomy was performed for selected patients in the present study. Another study compared simultaneous and staged pulmonary resection and found that the removal of nine of more segments increased significant risk of complications in simultaneous resections (11). As for lobectomy, because of its considerable invasiveness and high mortality, Mun et al. recommended that bilateral lobectomy should be performed *via* staged surgery instead of simultaneous surgery (21). It is worth noting that right lower lobectomy is the only significant predictor for the risk of short-term complications after bilateral pulmonary lobectomy (19). If right lower lobectomy is necessary, staged surgery should be given priority.

There was a mean difference of approximately 3 days between drain removal and hospital discharge. Considering that bilateral simultaneous surgery has a great impact on the pulmonary function of patients, and there is no mature experience to learn from, we extended the observation time after the removal of drainage tube, resulting in a longer average discharge time.

The present study had several limitations. First, it was a retrospective study with inherent selection bias. Second, the sample was small, with only 11 patients being reviewed. Last, no cases of staged surgery were used as a control group, as staged pulmonary surgery is almost never performed in our institution. A prospective multi-center study with a large sample is therefore required to verify the conclusions of the present study.

## Conclusion

Between January 2016 and August 2021, simultaneous bilateral pulmonary surgery was performed to treat 11 patients with bilateral pulmonary lesions including multiple primary lung cancer. It was found that simultaneous bilateral pulmonary surgery using VATS for the treatment of bilateral pulmonary lesions is safe and feasible and can therefore be considered after strict preoperative evaluation of the patient.

## Data availability statement

The original contributions presented in the study are included in the article. Further inquiries can be directed to the corresponding author.

## Ethics statement

The studies involving human participants were reviewed and approved by Third Affiliated Hospital, Navy Medical University ethics committee. The patients/participants provided their written informed consent to participate in this study.

## Author contributions

Conception and design of the research: XT and TP. Acquisition of data: JZ, WW. Analysis and interpretation of the data: JZ, HZ. Statistical analysis: JZ, ZS. Writing of the manuscript: XT, JZ. Critical revision of the manuscript for intellectual content: XT and TP. All authors contributed to the article and approved the submitted version.

## Conflict of interest

The authors declare that the research was conducted in the absence of any commercial or financial relationships that could be construed as a potential conflict of interest.

## Publisher’s note

All claims expressed in this article are solely those of the authors and do not necessarily represent those of their affiliated organizations, or those of the publisher, the editors and the reviewers. Any product that may be evaluated in this article, or claim that may be made by its manufacturer, is not guaranteed or endorsed by the publisher.
